# Stress and perceived health among primary care visitors in two corners of Europe: Scandinavia and Greece

**DOI:** 10.1186/s12942-020-00248-8

**Published:** 2020-12-04

**Authors:** Jenny Koppner, Marios Chatziarzenis, Tomas Faresjö, Elvar Theodorsson, Annika Thorsell, Staffan Nilsson, Ole Olsen, Åshild Faresjö

**Affiliations:** 1 Department of Health, Medicine and Caring Sciences/General Practice, Faculty of Health Sciences, Linköping University, Linköping, Sweden; 2 Elefsina Health Center, Thriasson General Hospital of Elefsina, Athens, Greece; 3 Department of Biomedical and Clinical Sciences/Clinical Chemistry, Faculty of Health Sciences, Linköping University, Linköping, Sweden; 4 Department of Biomedical and Clinical Sciences/Center for Social and Affective Neuroscience, Medicine, Linköping University, Linköping, Sweden; 5 Department of Health and Care Sciences, University of Tromsö, Tromsö, Norway; 6 Department of Health, Medicine and Caring Sciences/Public Health, Faculty of Health Sciences, Linköping University, Linköping, Sweden

**Keywords:** Stress, Cortisol, Depression, Anxiety, Perceived health, Economic crisis, Scandinavia, Greece

## Abstract

**Background:**

The global financial crisis emerging in 2008 struck Greece especially hard, whereas Scandinavian countries were less affected. This has created a unique opportunity to study the long-term effect of community stress on populations. Increasing frequencies of mental health issues and poorer perceived health among the Greek population have been reported. The physiological marker of long-term stress, cortisol in hair, is applied in this study together with measures of perceived health and stress, depression and anxiety. Our aim was to study self-reported and physiological stress, perceived health, including mental health, in the general population of Greece compared to Scandinavia, in order to assess long-term effects of the economic crisis on these parameters.

**Methods:**

A cross-sectional comparative study of adult (18–65 years) Primary Health Care visitors from semi-rural areas in Greece (n = 84) and Scandinavia (n = 140). Data collection was performed in 2012, and encompassed a questionnaire with a variety of health and stress indicators as well as hair samples for analyzes of cortisol levels.

**Results:**

The Greek sample reported significantly poorer overall health (p < 0.0001) than the Scandinavians and a significantly higher perceived stress (p < 0.0001). The Greeks were also less hopeful of the future (p < 0.0001), and to a larger extent fulfilled the HAD criteria for depression (p < 0.0001) and anxiety (p = 0.002). The strongest predictors explaining ill health in logistic regressions were being Greek (p = 0.001) and feeling hopeless about the future p = 0.001, OR = 6.00 (CI 2.10–14.88). Strong predictors in logistic regressions for high perceived stress were anxiety: high (p < 0.0001) and medium (p = 0.0001), as well as medium depression (p = 0.02).

**Conclusions:**

Greek adult Primary Health Care visitors perceived their health more negatively than the Scandinavians, including a higher presence of depression, anxiety, and a lower hope for the future. The Greeks also reported higher perceived stress, but this was not reflected in higher cortisol levels. The findings presented here, identify possible adverse long-term effects of the economic crisis in the examined Greek population that are not seen in the Scandinavian cohort. These differences may also be interpreted against the background of socio-cultural differences in the northern and south-eastern corners of Europe.

## Introduction

Mental and physical illness in a community are factors known to correlate with economic recession [1, 2]. In 2008, an international financial crisis emerged that engulfed southern Europe, hitting e.g. Greece especially hard [3]. In the years following the onset of the crisis, negative health consequences in the Greek population have been connected to perceived diminishing health, and increased prevalence rates of mental health problems including depression [4, 5]. Additionally, an increase in suicide rates has been recorded [6], and suicide attempts have been associated to increased hopelessness [7]. During the years of economic crisis in Greece it was found that young Greek adults reported higher perceived stress but had lower physiological stress than comparable young adults in Sweden [8].

Stress-related symptoms and disorders can commonly be seen in patients visiting Primary Health Care worldwide. These types of disorders have during the last decades become a focal point in a wide variety of health research as it constitutes an increasing global public health problem. This has also been recognized by the WHO, which states that “mental health problems and stress-related disorders are the biggest overall cause of early death in Europe” [9].

Self-reported health is an important and established public health indicator. Also, self-reports of perceived stress are an important indicator of stress-exposure [10]. For physiological short term or current stress, the steroid hormone cortisol is commonly measured [11–14]. The traditional methods for analyzing cortisol use blood, saliva, or urine, but these only indicate momentary stress, i.e. over a short time interval, and are also affected by the diurnal rhythm of cortisol release. In order to measure more long-term cortisol concentrations, a method using hair-samples has been developed and this method has now become a rather well-established tool in stress research [15]. Cortisol in hair reflects the activity of the HPA-axis as a mean value over a time period up to several months (1 cm of hair equals appr. 1 month). Up to date, research exploring possible associations between cortisol levels in hair and disease has found significant correlations of hair cortisol levels with e.g. mental disorders, chronic pain, and long-term unemployment [13, 16, 17].

Here, set against the background of differences in impact of the 2008 global financial crisis on southern and northern Europe, we have conducted a comparative study of stress and perceived health among Primary Health Care visitors in two corners of Europe, Scandinavia and Greece. Our working hypothesis being that the Greek population would report lower perceived health and higher perceived stress with corresponding differences in cortisol levels in hair than the Scandinavian population.

### Aim of the study

In this study we aimed to analyze self-reported and physiologically measured stress, mental health problems, and perceived health in adult visitors at Primary Health Care centers in Greece compared to equivalent Primary Health Care visitors in Scandinavia, i.e. Sweden and Norway. The aim was to identify long-term effects persisting in the populations four years after the onset of the global financial crisis of 2008, and further to identify differences between the study-populations in the above measures.

## Methods

### Participants

This cross-sectional study included adults of working age (18–65 years) visiting semi-urban Primary Health Care Centers (PHC) during 2012, one PHC in Greece and three in Scandinavia (two in Sweden and one in Norway). The participants were recruited consecutively during a visit to the PHC. However, since PHC visitors as a group normally consists of a high percentage of elderly and chronically ill persons [18], a random selection of elderly visitors was used to avoid an overrepresentation of this age-group in the sample. Since the sampling method in this study was consecutive and random, no data was recorded about dropouts or how many and/or why people chose not to participate. The recruiting personnel’s estimation was that approximately 50% declined, evenly spread among ages, sexes, and sites. Reasons, when such were given, for not wanting to participate were e.g. patient being called into a medical appointment or the patient not wanting to give a hair sample.

Prior to data collection, power was calculated as n = 58 at each study site to reach a significance level of 0,05 and a power of 80%. The total number of participants included were n = 84 in Greece and n = 140 in Scandinavia. Participants at the three Scandinavian sites were pooled into one group. Characteristics of the participants are described in Table 1. Noticeably, some questions regarding overall characteristics presented in Table 1 were not answered by all participants, including: Gender (Scandinavians), Age, Perceived health, Hope for the future, Perceived stress (Scandinavians), HAD depression (Scandinavians), HAD anxiety (Scandinavians). Reasons for non-responses were not given. The largest drop-out, 9/140, was for Age among the Scandinavian population.

**Table 1:** Characteristics of different variables for the Greek and Scandinavian samples

Variables	Greek N = 84 n (%)	Scandinavian N = 140 n (%)	p-value
Gender Male Female	18 (21) 66 (79)	32 (23) 108 (77)	0.87
Age^a^ 18-29 years 30-49 years 50-65 years	14 (17) 48 (58) 21 (25)	27 (21) 56 (43) 48 (37)	0.09
Smoker Yes	31 (37)	20 (15)	< 0.0001
Unemployed Yes	17 (21)	6 (4)	< 0.0001
Longstanding illness Yes	20 (25)	40 (29)	0.28
Medication with glucocorticoids Yes	4 (5)	8 (6)	0.99
Serious life-events Yes	55 (65)	36 (26)	< 0.0001
Perceived health Good Bad	29 (35) 53 (65)	110 (83) 23 (17)	< 0.0001
Perceived stress Low Moderately High/very high	7 (8.3) 39 (46.4) 38 (45.2)	40 (29.2) 63 (46.0) 34 (24.8)	< 0.0001
Hope for the future Hopeful Hopeless	43 (52) 39 (48)	131 (97) 4 (3)	< 0.0001
HAD depression None Moderate High	46 (55) 22 (26) 16 (19)	123 (87) 11 (8) 2 (2)	< 0.0001
HAD anxiety None Moderately High	37 (44) 22 (26) 25 (30)	90 (66) 29 (21) 18 (13)	0.002

^a^Missing data for Age: Greece n = 1, Scandinavian n = 9

### Procedures and measures

Sociodemographic variables were collected using a questionnaire divided into three parts that included validated and previously tested questions measuring variables including: age, sex, employment, self-report of long-standing chronic illness, regular medication, and exposure to serious life events (e.g. divorce, unemployment, surgery, economic problems, serious illness or death in the family). Possible confounders were also included: regular medication with glucocorticoids, colored or permed hair, and smoking. The participants were additionally asked to self-estimate their general health as well as hope for the future. A Visual Analogue Scale (VAS) was used for self-estimated health (range: very bad–very good), hope for the future (range: hopeless–hopeful), and severity of serious life event (range: not at all stressful–very stressful). Each of these measures was then divided into five categories of increasing severity, and then further dichotomized for illustrative purposes. Included in the questionnaire was also the Hospital Anxiety and Depression Scale (HAD) [19], a diagnostic tool used in clinical practice, and the Perceived Stress Scale (PSS) [10]. For PSS the 10-item scale was chosen over the 14-item scale since it has slightly lower variance and better reliability [20], and in order to limit the number of questions included in order to increase compliance. Greek, Swedish and Norwegian established translations were used for both PSS and HAD [21–23]. When analyzing the results from PSS and HAD, each scale was divided into subgroups; HAD depression and HAD anxiety, respectively, were divided into three groups following the clinical cut-offs [28]. PSS, as it has no clinical cutoffs, was divided into four equal groups: 0-10p (low), 11-20p (medium), 21-30p (high), and 31-40p (very high); the high and very high scores were collapsed into one group for analyses [13].

Hair samples were collected from the vortex area of the head. Extraction and analysis of cortisol levels in hair was done using a competitive radioimmunoassay (RIA) of methanol extracts from hair samples that were frozen in liquid nitrogen and mechanically pulverized according to previously described procedure by Karlén et al. [24]. In this study, no hair sample was shorter than 3 cm in length, and all participants donated enough hair volume for analyses.

### Statistical analyses

All statistical analyses were performed using The Statistical Package for the Social Sciences (SPSS ver. 24.) software (Chicago, IL, USA). Spearman’s correlation was used for bivariate testing and associations between variables. Mann–Whitney analyses were performed to analyze differences between sites and relevant variables. Independent variables univariately statistically significant were included in binary logistic regression analyses to estimate the odds ratio (OR) with 95% confidence intervals (CI). Three models were created where the statistically significant factors related to the dependent factors perceived health and perceived stress from the univariate model were brought to the final model which was additionally adjusted for possible confounding factors. A p-value of p ≤ 0.05 was considered statistically significant.

## Results

### Overall characteristics of the sample populations

The sex distribution was quite equal between the two sample-populations with an overall excess of women in both sample-populations. Age-wise, the majority of participants at all sites were in the age group 30–49 years (Table 1). The number of smokers as well as unemployment rate were both significantly higher among the Greeks compared to the Scandinavians (p < 0.0001, both measures), and a higher percentage of Greeks reported exposure to serious life-events (p < 0.0001). There were no differences between the samples on self-reported longstanding illness (p = 0.277) or medication with glucocorticoids (p = 0.99).

**Fig. 1:**
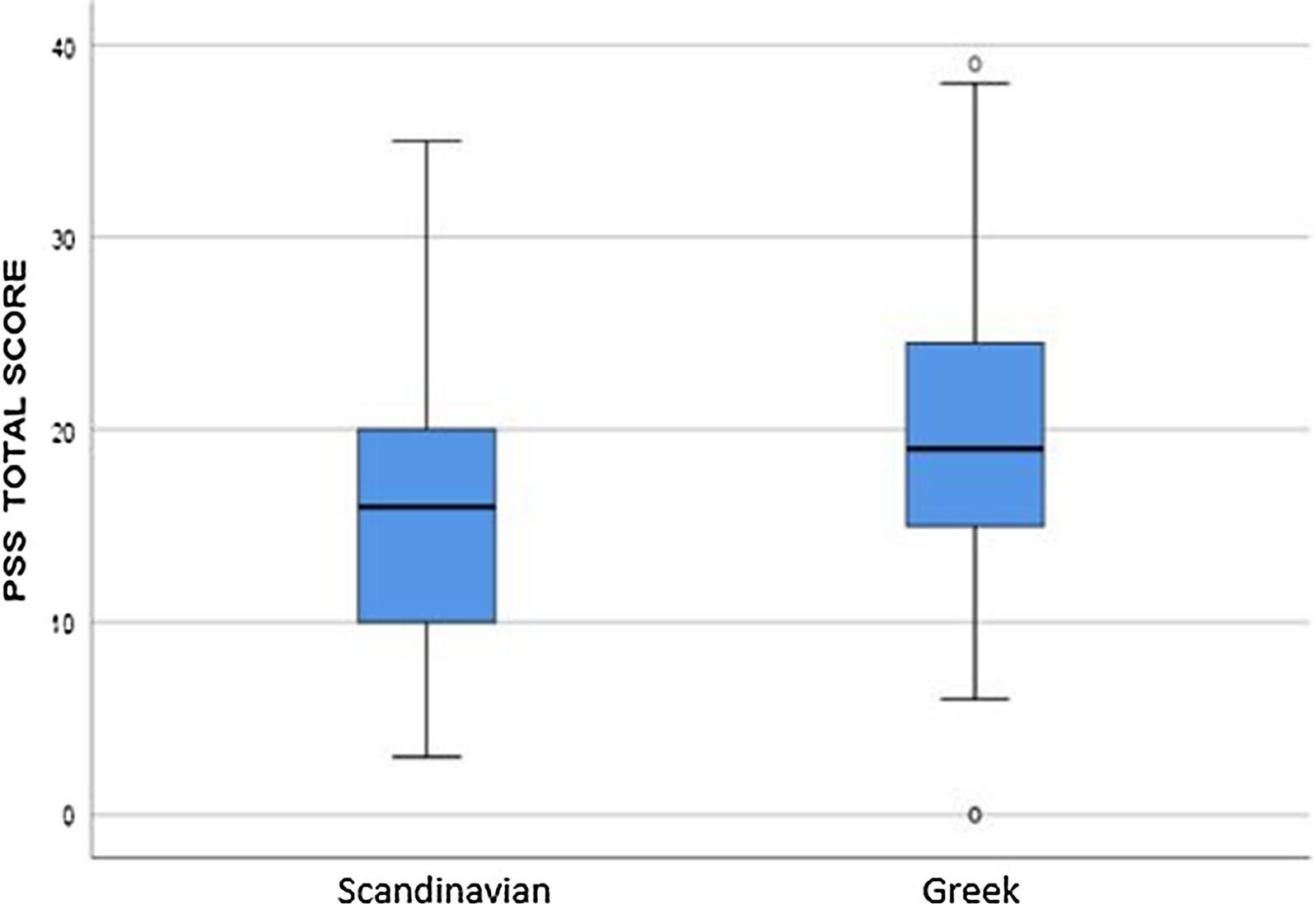
Perceived stress as measured by PSS in Scandinavian and Greek participants. The mean PSS score was 19.87 (median = 19, IQR = 10) for the Greeks and 15.73 (median = 16, IQR = 11) for the Scandinavians, p < 0.0001

The Greek sample generally displayed higher scores on negative health- and stress variables as they reported significantly poorer perceived health (p < 0.0001; Table 1) and higher perceived stress (p < 0.0001; Table 1, Fig. 1) compared to the Scandinavian population.

Furthermore, the Greeks displayed less hope for the future (p < 0.0001), and to a larger extent fulfilled the HAD criteria for depression (p < 0.0001) and anxiety (p = 0.002) than the Scandinavian sample (Table 1).

### Hair cortisol

**Fig. 2:**
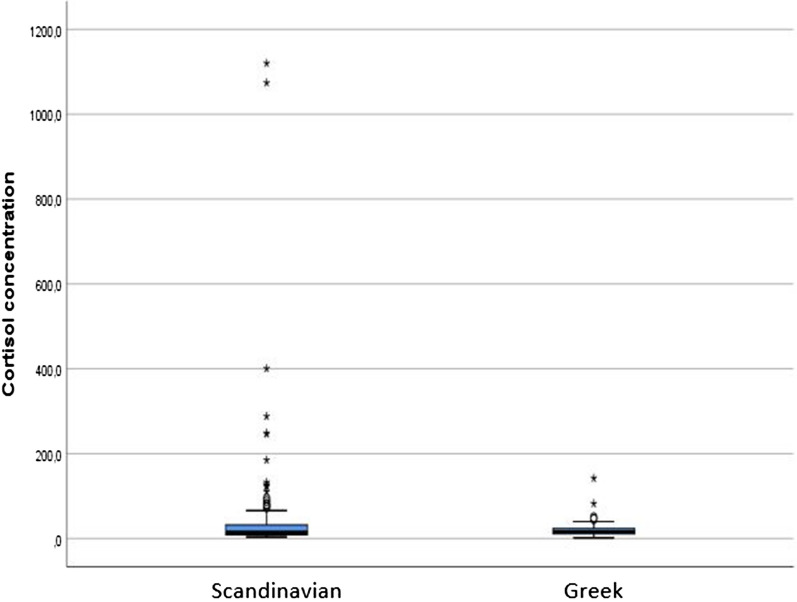
The distribution of cortisol concentration in hair among Greek and Scandinavian participants. For the Greeks, the median cortisol level was 15.6 (IQR = 12.9) and for the Scandinavians the median cortisol level was 14.6 (IQR = 23.8). These differences were not statistically significant (p = 0.80)

The distribution of hair cortisol concentration among the participants is shown in Fig. 2.

### Factors associated with lower perceived health and high perceived stress

Univariate analyses of factors possibly associated with perceived health are displayed in Table 2, and those possibly associated to perceived stress are shown in Table 3. For both outcomes, significant correlations between outcome and HAD depression, outcome and HAD anxiety, as well as outcome and PSS were shown. In the total population there was e.g. a positive correlation between PSS and previous experience of serious life event (p < 0.0001) self-reported low health (p < 0.0001), HAD anxiety (p < 0.0001) and HAD depression (p < 0.0001). Furthermore, there were no significant associations between perceived health or perceived stress and hair cortisol levels, as shown in Tables 2 and 3.

**Table 2:** Correlations between different variables and perceived health

Variable	Total population N = 224		Greece N = 84		Scandinavia N = 140	
	r-value	p-value	r-value	p-value	r-value	p-value
Sex	− 0.070	0.31	0.064	0.57	− 0.187	0.03
Age	− 0.176	0.001	− 0.219	0.05	− 0.140	0.12
Unemployment	0.023	0.73	− 0.160	0.15	0.023	0.73
Smoking	− 0.284	< 0.0001	− 0.261	0.02	0.097	0.27
Longstanding illness	− 0.133	0.05	− 0.487	< 0.0001	0.140	0.11
Self-reported stress	0.226	0.001	− 0.157	0.16	0.293	< 0.0001
Serious life events	0.147	0.03	− 0.284	0.01	0.124	0.16
Self-reported economic crisis	0.112	0.10	− 0.322	0.03	0.119	0.17
Hope for the future	0.475	< 0.0001	− 0.289	< 0.009	0.385	< 0.0001
HAD: Anxiety	0.165	0.002	− 0.114	0.28	0.283	0.001
HAD: Depression	0.113	0.003	− 0.255	< 0.05	0.370	< 0.0001
Cortisol value	− 0.084	0.32	0.093	0.41	− 0.024	0.78

r-value measured by Spearman’s rho

**Table 3:** Correlations between different variables and Perceived Stress levels

Variable	Total population N = 224		Greece N = 84		Scandinavia N = 140	
	r-value	p-value	r-value	p-value	r-value	p-value
Sex	− 0.030	0.66	0.065	0.55	− 0.092	0.28
Age	− 0.124	0.007	− 0.033	0.77	− 0.151	0.08
Unemployment	0.107	0.11	0.157	0.15	0.040	0.97
Smoking	0.043	0.53	− 0.065	0.56	0.016	0.85
Longstanding illness	0.045	0.51	0.095	0.40	− 0.161	0.61
Serious life events	0.401	< 0.0001	0.427	< 0.0001	− 0.014	0.87
Self-reported health	0.234	< 0.0001	− 0.157	0.16	0.293	0.001
Self-reported economic crisis	0.288	0.0001	0.269	0.01	0.071	0.41
Hope for the future	0.014	0.84	− 0.472	< 0.0001	0.239	< 0.005
HAD: Anxiety	0.585	< 0.0001	0.644	< 0.0001	0.517	< 0.0001
HAD: Depression	0.501	< 0.0001	0.510	< 0.0001	0.406	< 0.0001
Cortisol value	− 0.012	0.86	− 0.013	0.91	0.094	0.30

r-value measured by Spearman’s rho

A set of logistic regressions were performed to elaborate possible factors associated with respectively low perceived health (Table 4) and high perceived stress (Table 5). The strongest predictors of ill health were being Greek (p = 0.001, OR 3.94 (CI 1.81–8.60)) and feeling hopeless about the future (p = 0.001, OR 6.00 (CI 2.10–14.88)). Furthermore, Greek individuals who had experienced previous serious life events or were smokers were found to report poorer health (Serious life events: p = 0.04, OR 2.12 (CI 1.05–4.27); smokers: p = 0.05, OR 2.37 (CI 1.00–5.62)). Additionally, middle-aged people (50–65) as a group reported significantly poorer health p = 0.03, OR 0.32 (CI 0.11–0.92).

**Table 4:** Logistic regression of possible factors explaining low perceived health

Factors	Model 1		Model 2		Model 3		Final model	
	OR (95% CI)	p-value	OR (95% CI)	p-value	OR (95% CI)	p-value	OR (95% CI)	p-value
Scandinavian	Reference		Reference		Reference		Reference	
Greek	5.51 (2.15–14.14)	< 0.0001*	8.07 (3.96–16.50)	< 0.0001*	8.57 (4.06–18.09)	< 0.0001*	3.94 (1.81–8.60)	0.001*
Female	Reference		Reference		Reference		Reference	
Male	0.69 (0.27–1.75)	0.44	0.62 (0.26–1.51)	0.30	0.65 (0.28–1.56)	0.34	0.71 (0.29–1.75)	0.45
Age (18–29Y)	Reference		Reference		Reference		Reference	
Age (30–49 y)	1.34 (0.50–3.55.)	0.56	1.54 (0.63–3.78)	0.34	1.66 (0.68–4.09)	0.26	1.24 (0.51–3.04)	0.64
Age (50–65 Y)	0.35 (0.16–1.05)	0.06	0.39 (0.14–1.19)	0.07	0.48 (0.16–1.47)	0.20	0.32 (0.11–0.92)	0.03*
Employed	Reference		–	–	–	–	–	–
Unemployed	1.21 (0.50–2.94)	0.70	–	–	–	–	–	–
Not smoking	Reference		–	–	–	–	Reference	
Smoker	2.24 (1.00–5.39.76)	0.07					2.37 (1.00–5.62)	0.05*
No experience of economic crisis	Reference		–	–	–	–	–	–
Experience of conomic crisis	0.50 (0.15–1.46	0.20	–	–	–	–	–	–
No Serious life events	Reference		–	–	–	–	Reference	
Yes serious life events	1.00 (0.37–2.39)	0.90	–	–	–	–	2.12 (1.05–4.27)	0.04*
Hope for the future	Reference		–	–	–	–	Reference	
Hopeless about the future	5.39 (1.97–14.75)	0.001*	–	–	–	–	6.00 (2.10–14.88)	0.001*
Cortisol medium	–	–	Reference		_	–	–	–
Cortisol low	–	–	2.71 (0.31–23.76	0.37				
Cortisol high	–	–	3.50 (0.40–1.43)	0.26	–	–	–	–
Low perceived stress	–	–	Reference		–	–	–	–
High perceived Stress	–	–	1.76 (0.85–3.65)	0.13	–	–	–	–
No longstanding illness	–	–	–	–	Reference			
Yes longstanding illness	–	–	–	–	0.68 (0.30–1.60)	0.38	–	–
No Anxiety	–	–	–	–	Reference			
Anxiety medium	–	–	–	–	1.30 (0.55–3.06)	0.55	–	–
Anxiety high	–	–	–	–	2.02 (0.67–6.05)	0.21		
No Depression	–	–	–	–	Reference		–	–
Depression medium	–	–	–	–	0.95 (0.33–2.779	0.93		
Depression high	–	–	–	–	0.66 (0.15–2.90)	0.58	–	–

Model 1 includes background factors and socioeconomic and psychosocial factors, model 2 includes background factors and stress indicators, model 3 includes background factors and indicator of illness. The final model only includes the background factors and significant indicators from the models. The model was significant, p-value 0.0001, Cox&Snell R Square 0.316 and Nagelkerke R Square 0.435

**Table 5:** Logistic regression of possible factors explaining high perceived stress

Factors	Model 1		Model 2		Model 3		Final model		
	OR (95% CI)	p-value	OR (95% CI)	p-value	OR (95% CI)	p-value	OR (95% CI)		p-value
Scandinavian	Reference		Reference		Reference			Reference	
Greek	1.20 (0.50–2.90)	0.70	1.62 (0.78–3.36)	0.20	1.01 (0.46–2.22)	0.98		0.84 (0.36–1.95)	0.68
Female	Reference		Reference		Reference			Reference	
Male	0.84 (0.37–1.89)	0.67	0.89 (0.40–1.96)	0.78	0.80 (0.34–1.90)	0.60		0.77 (0.33–1.81)	0.55
Age (18–29 Y)	Reference		Reference		Reference			Reference	
Age (30–49 y)	1.01 (0.43–2.66)	0.90	1.38 (0.54–3.52)	0.45	1.61 (0.54–4.83)	0.40		1.30 (0.46–3.64)	0.62
Age (50–65 Y)	0.66 (0.25–1.73)	0.40	1.39 (0.64–3.00)	0.40	1.86 (0.75–4.61)	0.20		1.55 (0.70–3.52)	0.30
Employed	Reference		–	–	–	–		–	–
Unemployment	1.00 (0.42–2.04)	0.84	–	–	–	–		–	–
Not smoking	Reference		–	–	–	–		–	–
Smoker	1.25 (0.57–2.76)	0.60							
No experience of economic crisis	Reference		–	–	–	–		–	–
Experience of conomic crisis	1.60 (0.60–4.20)	0.35	–	–	–	–		–	–
No Serious life events	Reference		–	–	–	–		Reference	
Yes serious life events	2.18 (1.00–4.76)	0.05*	–	–	–	–		1.64 (0.77–3.52)	0.21
Hope for the future	Reference		–	–	–	–		–	–
Hopeless about the future	1.00 (0.37–2.22)	0.84	–	–	–	–		–	–
Cortisol medium	–	–	Reference		_	–		–	–
Cortisol low	–		1.27 (0.60–2.47)	0.58	–	–		–	–
Cortisol high	–	–	1.67 (0.39–7.17)	0.41	–	–		–	–
Good perceived health	–	–	Reference		–	–		–	–
Bad perceived health	–	–	1.70 (0.81–3.53)	0.16	–	–		–	–
No longstanding illness	–	–	–	–	Reference				
Yes longstanding illness	–	–	–	–	1.32 (0.57–3.08)	0.51		–	–
No Anxiety	–	–	–	–	Reference			Reference	
Anxiety medium	–	–	–	–	5.06 (2.20–11.63)	0.0001*		4.90 (2.10–11.31)	0.0001*
Anxiety high	–	–	–	–	5.61 (1.95–16.17)	< 0.001*		3.79 (1.80–7.96)	< 0.0001*
No Depression	–	–	–	–	Reference			Reference	
Depression Medium	–	–	–	–	3.58 (1.29–9.94)	0.01*		3.32 (1.20–9.29)	0.02*
Depression high	–	–	–	–	1.56 (0.38–6.43)	0.54		1.49 (0.36–6.16)	0.21

Model 1 includes background factors and socioeconomic and psychosocial factors, model 2 includes background factors and stress indicators, model 3 includes background factors and indicator of illness. The final model only includes the background factors and significant indicators from the models. The model was significant, p-value 0.0001, Cox& Snell 0.204 and Nagelkerke 0.300

The strongest predictors in logistic regressions for high perceived stress were high or medium levels of measured anxiety (OR 3.79; p < 0.0001 and OR 4.90; p = 0.0001, respectively). Also, depression of intermediate range was a significant predictor of high perceived stress (OR3.32; p = 0.02). Individuals with either low or high cortisol levels as well as those that reported poor health had elevated risks for reports of high stress, but these were not statistically significant.

## Discussion

Our main finding in this study was that adult Greek PHC visitors had lower self-reported health, more symptoms of depression and anxiety, higher perceived stress, and lower hope for the future than their Scandinavian peers, consistent with our hypothesis. Important is also that more Greek participants fulfilled the criteria for depression and anxiety with the HAD questionnaire than their Scandinavian counterparts. These findings are consistent with a previous study comparing health and stress among Greek and Swedish young adults [8]. Notably, in the present study there were no significant differences in physiologically measured stress between Greeks and Scandinavians, contrary to what we hypothesized. The results suggest that the Greeks feel more stressed but that the perception of stress at this magnitude is not mirrored as an increased activity of the HPA-axis. However, we also consider that the population-size might need to be expanded in order to detect a significant difference in cortisol levels among PHC visitors, since they are a heterogenous group both with regards to age and health status, compared to, for example, the university students that constituted the population in our previous study [8]. The overall differences in perceived health and stress we have found might reflect the general exposure to long-lasting community stress due to the after-effects of the economic crisis in Greece. This consequential association requires another study design, but the reasoning behind it is strengthened by the previous reports of increasing suicide rates [6] and mental health problems [4, 5] during the years of a financially challenged Greece.

The participants in the present study were ordinary primary health care visitors, a mixture of all adult age-groups with a predominance of middle-aged to older, and presumably more ill, patients. Whether age alters cortisol levels is not fully determined [25, 26], but the older PHC visitors might be more affected by the economic crisis as they probably have more family responsibilities making them more vulnerable to financial strain, unemployment, and other austerity in health- and welfare [4], inducing higher perceived stress and poorer health. On the other hand, with age comes life experience and older PHC visitors might have better developed coping strategies that would reduce stress. A study among elderly in Israel concluded that personal resources and use of appropriate coping behaviors enabled elderly people to control their well-being even in the presence of declined health or function [27]. Health behavior and not least health care service utilization is highly variable between northern and southern Europe. Living in different social and cultural environments, e.g. Scandinavia and Greece, could also have impact on perceptions of health and stress [28]. However, important to note here is that this cross-sectional study design cannot determine causality.

A strength of this study is the unique opportunity to study possible signs of deteriorated health and community stress in a PHC population from Greece, a country that has suffered from several years of financial crisis compared to a similar population in Scandinavia without this burden. Of course, there are differences between the populations regarding socio-cultural factors. Public health, health behavior, and health care utilization are highly variable across the European continent. This is most evident when comparing north-western and south-eastern Europe. These geographical corners of Europe also differ in other health-related aspects. Typical for the countries in the south-eastern Mediterranean area, like Greece, is the classical Mediterranean food and nutrition, the socio-culture traditions with tight social and family bonds and outdoor living which is still found in Greece [29–32]. Scandinavian countries like Norway and Sweden represent welfare states that offers their citizen’s public institutions like; kindergartens, elderly home care, free schools including university education and health care insurance for all. The welfare states also aim to reduce income inequalities by economic transfers between rich and poor.

Socio-cultural differences in health beliefs is illustrated in a study of sense of coherence between a Swedish and a Cretan population. It was found that Cretan men had significantly higher score of sense of coherence than Swedish men but, on the other hand, Cretan women had significantly lower scores than Swedish women [33]. In a study of the disease Irritable Bowel Syndrome (IBS), it was found that individuals with the disease but living in different cultural environments, i.e. Sweden and Greece, perceived their disease differently, and the disease affected their everyday life and quality of life differently. The Greek women with IBS had more severe symptoms, and scored lower on all measures of perceived health, well-being and quality of life than the Swedish women [28].

Another strength in this study is the use of PHCs for recruiting participants as it enabled us to easily reach a wide variety of participants of both sexes and all ages. However, though all samples come from patients in primary care, one must keep in mind that Scandinavian countries have well-developed primary health care centers accessible for all, while Greece just recently started to establish a primary health care organization in urban areas [34]. This disparity is also evident in organizational differences, as well as in care seeking patterns, in northern and southern Europe. In Scandinavia, a citizen might visit primary care on average once a year, while a Greek citizen might visit their primary care up to six times per year [35]. Prescribing patterns by general practitioners and recipe-renewals also differ across sites in Europe, resulting in more or less frequent health care visits [36]. This could possibly be due to different burden of disease between the populations and thus skew the results, but also possibly due to differences in socio-cultural behavior. However, we found no significant difference with regards to prevalence of longstanding illness between the populations, although smoking was significantly more common in Greece, which may suggest a higher prevalence of smoking-related diseases.

As predicted, the Greek population suffered from lower perceived health and stress, as well as higher levels of experienced anxiety and depression, but their cortisol levels were not significantly different from the Scandinavians. Therefore, the physical stress-response is unlikely to have caused a higher degree of somatic illness, at least not at the time for the data collection. A possible explanation is that positive socio-cultural factors such as higher sense of coherence and Mediterranean diet may counteract biological stress and subsequent longstanding somatic disease. Using validated and established questionnaires like HAD and PSS is a strength of this study, enabling us to relate the obtained results to previous studies, as well as being globally used and accepted methods of evaluation.

A limitation of the study may be the sample size, but the study has nonetheless an acceptable statistical power according to our power calculations. An obvious limitation in studies where hair cortisol is measured is “the natural drop-out” among men. This was seen at all sites and is a general limitation of the measurement of cortisol in hair. However, the hair-cortisol analysis method allows for a measurement that is independent of the diurnal changes in cortisol and gives a long-term estimate of cortisol levels. Furthermore, the RIA used here is considered both robust and sensitive [37].

## Conclusions

We found that adult Greek PHC visitors experienced significantly poorer perceived health and a higher level of perceived stress, including more symptoms of depression and anxiety as well as lower hope for the future, than the Scandinavian sample. The results of this study might reflect the long-term effects of the 2008 economic crisis in Greece, as well as differences in social and cultural tradition including health behavior and health care service utilization between the sites. Contrasting these two corner sites of Europe provides further understanding of how community stress exposure triggered by economic crisis could affect health and stress in the society.

## Data Availability

All data is available and can be obtained from the corresponding author on request.
